# Correction to: 114. Long-term Protection Against Herpes Zoster (HZ) by the Adjuvanted Recombinant Zoster Vaccine (RZV): Interim Efficacy, Immuno and Safety Results at Approximately 10 Years after Initial Vaccination

**DOI:** 10.1093/ofid/ofad251

**Published:** 2023-05-11

In the original publication of this abstract, [Strezova A, Diez-Domingo J, Al Shawafi K et al. 114. Long-term Protection Against Herpes Zoster (HZ) by the Adjuvanted Recombinant Zoster Vaccine (RZV): Interim Efficacy, Immuno and Safety Results at Approximately 10 Years after Initial Vaccination, Open Forum Infectious Diseases, Volume 9, Issue Supplement_2, December 2022, ofac492.192 https://doi.org/10.1093/ofid/ofac492.192], a reference to the Zoster-049 Study Group was missing from the author byline, and another location for GSK was missing from the affiliations list. These have been inserted into the version of the abstract currently available online, and the authors regret this error.

